# Prognostic Impact and Clinical Implications of Adverse Tumor Grade in Very Favorable Low- and Intermediate-Risk Prostate Cancer Patients Treated with Robot-Assisted Radical Prostatectomy: Experience of a Single Tertiary Referral Center

**DOI:** 10.3390/cancers16112137

**Published:** 2024-06-04

**Authors:** Antonio Benito Porcaro, Alberto Bianchi, Sebastian Gallina, Andrea Panunzio, Alessandro Tafuri, Emanuele Serafin, Rossella Orlando, Giovanni Mazzucato, Paola Irene Ornaghi, Francesco Cianflone, Francesca Montanaro, Francesco Artoni, Alberto Baielli, Francesco Ditonno, Filippo Migliorini, Matteo Brunelli, Salvatore Siracusano, Maria Angela Cerruto, Alessandro Antonelli

**Affiliations:** 1 Department of Urology, University of Verona, Azienda Ospedaliera Universitaria Integrata, 37129 Verona, Italy; drporcaro@yahoo.com (A.B.P.); alberto.bianchi@aovr.veneto.it (A.B.); sebastian.gallina@univr.it (S.G.); panunzioandrea@virgilio.it (A.P.); emanuele.serafin@univr.it (E.S.); rossella.orlando@univr.it (R.O.); giovanni.mazzucato@univr.it (G.M.); paolairene.ornaghi@univr.it (P.I.O.); francesco.cianflone@univr.it (F.C.); francesca.montanaro@univr.it (F.M.); francesco.artoni@univr.it (F.A.); alberto.baielli@univr.it (A.B.); filippo.migliorni@aovr.veneto.it (F.M.); mariaangela.cerruto@univr.it (M.A.C.); alessandro.antonelli@univr.it (A.A.); 2 Department of Urology, Vito Fazzi Hospital, 73110 Lecce, Italy; aletaf@hotmail.it; 3 Department of Urology, Rush University Medical Center, Chicago, IL 60612, USA; 4 Department of Pathology, University of Verona, Azienda Ospedaliera Universitaria Integrata, 37129 Verona, Italy; matteo.brunelli@univr.it; 5 Department of Life, Health and Environmental Sciences, University of L’Aquila, 67100 L’Aquila, Italy; salvatore.siracusano@univaq.it

**Keywords:** prostate cancer, low-risk prostate cancer, intermediate-risk prostate cancer, robot-assisted radical prostatectomy (RARP), adverse pathology outcomes, tumor upgrading, tumor upstaging, progression of prostate cancer

## Abstract

**Simple Summary:**

The prognosis of PCa at diagnosis is assessed by classification systems that aim to group homogenous sets of patients. However, great heterogeneity exists within these risk groups, in terms of prognostic factors and treatment options. The aim of this study was to assess the prognostic impact and predictors of adverse tumor grade in very favorable low- and intermediate-risk prostate cancer patients treated with robot-assisted radical prostatectomy at a high-volume tertiary referral center.

**Abstract:**

Objectives: To assess the prognostic impact and predictors of adverse tumor grade in very favorable low- and intermediate-risk prostate cancer (PCa) patients treated with robot-assisted radical prostatectomy (RARP). Methods: Data of low- and intermediate PCa risk-class patients were retrieved from a prospectively maintained institutional database. Adverse tumor grade was defined as pathology ISUP grade group > 2. Disease progression was defined as a biochemical recurrence event and/or local recurrence and/or distant metastases. Associations were assessed by Cox’s proportional hazards and logistic regression model. Results: Between January 2013 and October 2020, the study evaluated a population of 289 patients, including 178 low-risk cases (61.1%) and 111 intermediate-risk subjects (38.4%); unfavorable tumor grade was detected in 82 cases (28.4%). PCa progression, which occurred in 29 patients (10%), was independently predicted by adverse tumor grade and biopsy ISUP grade group 2, with the former showing stronger associations (hazard ratio, HR = 4.478; 95% CI: 1.840–10.895; *p* = 0.001) than the latter (HR = 2.336; 95% CI: 1.057–5.164; *p* = 0.036). Older age and biopsy ISUP grade group 2 were independent clinical predictors of adverse tumor grade, associated with larger tumors that eventually presented non-organ-confined disease. Conclusions: In a very favorable PCa patient population, adverse tumor grade was an unfavorable prognostic factor for disease progression. Active surveillance in very favorable intermediate-risk patients is still a hazard, so molecular and genetic testing of biopsy specimens is needed.

## 1. Introduction

As of today, the prognosis of PCa at diagnosis is evaluated using classification systems designed to categorize homogeneous sets of patients [1,2,3]. The two main prognostic systems for PCa are the European Association of Urology (EAU) and National Comprehensive Cancer Network (NCCN) guidelines. Even though they reference D’Amico’s risk classes, they are not entirely comparable [1,2]. Although parameters defining the low-risk class are consistent for NCCN and EAU, the former considers a very favorable subgroup, that less than 50% of the cores collected at biopsies are positive cores with cancer involvement not exceeding 50% of each core, clinical stage cT1c (non-palpable tumors) and PSA density (PSAD) less than 0.15 ng/mL/mL [1,2]. Even though the palpability of prostate tumors at presentation is an important parameter for clinical staging, palpable tumors involving both lobes of the prostate (cT2c) are categorized in the intermediate-risk class by NCCN, whereas EAU classifies such cancers as belonging to the high-risk category. However, clinical stage cT1c is included in both systems’ categories, denoting low and intermediate risk [1,2]. In addition, the latter considers a favorable intermediate-risk class to be a subset defined by PSA levels between 10–20 ng/mL or tumor grade group 2, according to the International Society of Urologic Pathology (ISUP) system, or clinical stage cT2b-2c and a less than 50% percentage of biopsy positive cores (BPC); nevertheless, patients may present with PSA < 10 ng/mL as well ISUP grade group 2, thus representing a very favorable subgroup, but are still included in the aforementioned group [1,2].

Thus, favorable low- and intermediate-risk classes are very important prognostic subgroups that promote active surveillance (AS) with eventual delayed treatment [1,2]. However, radical prostatectomy (RP) and radiation therapy (RT) are pursuable strategies in patients with favorable low and intermediate PCa [1,2]. On the other hand, the issue of tumor misclassification, particularly concerning upgrading and upstaging patterns, persists as a challenge today, with an increased risk of disease progression (which includes undifferentiated (ISUP ≥ 3) and non-organ-confined tumors) [1,2,3,4,5,6,7]. Despite this, until prognostic molecular markers are integrated, there is a pressing need to improve conventional clinical predictors of disease progression in very favorable low and intermediate categories to reduce treatment-related regret [8]. So far, early PCa may encompass highly favorable subsets within the low- and intermediate-risk categories defined by the EAU, including patients with PSA levels < 10 ng/mL, BPC < 50%, and non-palpable tumors (cT1c), but differing only in the ISUP grade group, which is 1 for the former and 2 for the latter, respectively.

In this study, we aimed to evaluate the influence of unfavorable tumor grade (ISUP grade group > 2) on PCa progression after RARP in a very favorable PCa population with low/intermediate risk-class features, presenting with PSA < 10 ng/mL, BPC < 50%, clinical stage cT1c (non-palpable tumors) and including biopsy ISUP grade groups 1 and 2. The secondary endpoint was to assess clinical and pathological predictors of unfavorable tumor grade.

## 2. Materials and Methods

### 2.1. Data Acquisition, Patient Selection, Parameter Assessment

The Institutional Review Board granted approval for the study, which obtained informed consent from all participants. From our prospectively maintained institutional prostate cancer database, we extracted and retrospectively analyzed all patients (n = 1143) who underwent RARP—as per institutional protocol—between January 2013 and October 2020. Out of these, 901 subjects with both follow-up data and relevant clinical and tumor data were considered for further selection. Ultimately, 479 were classified as intermediate risk and included in the analysis.

Moreover, prostate-specific antigen (PSA; ng/mL), age (years), body mass index (BMI; kg/m^2^), prostate volume PV (mL, both measured by the trans-rectal approach and by TRUS) and BPC (%), and PSAD (ng/mL/cc) were assessed. Biopsies conducted in other centers were evaluated for the number of cores obtained and tumor grade. Clinical staging was determined in accordance with the 2017 version of the TNM system (8th edition). Also, the American Society of Anesthesiologists (ASA) classification system was recorded [9]. RARP were carried out by proficient surgeons. Extended pelvic lymph node dissection (ePLND) was conducted in adherence to established guidelines [1,2]. A standardized anatomical template was employed to package individually dissected lymph nodes, comprised bilaterally of external iliac, internal iliac plus obturator, Marcille’s common iliac, and Cloquet’s nodal stations; also, peritoneal reconfiguration was employed to reduce the risk of lymphoceles in select cases [10,11]. Specimens, including the prostate and potentially dissected lymph nodes, were immersed in formalin prior to anatomopathological examination. Prostate glands were weighed, and the ISUP grading system was employed to establish tumor grading [1,2]. Tumor load (TL) was defined as the percentage of prostate tissue affected by cancer. Specifically, our dedicated uropathologist assessed TL by visually estimating all glass slides, with microscopically identifiable carcinoma foci circled using a marked pen, in accordance with ISUP guidelines [12]. Surgical margins (SM) were deemed positive, as per guidelines, and classified as focal and non-focal based on the linear extent [1]. Excised lymph nodes were enumerated and examined for cancer infiltration. Surgical specimens were staged in accordance with the 2017 version of the TNM system (8th edition) [1,2]. Perioperative outcomes included were operating time, estimated intraoperative blood loss, nerve-sparing procedures, surgeon experience (high and low volume), length of hospital stay (LOHS), and readmission to the hospital post-discharge. The Clavien–Dindo system was employed to assess postoperative complications, which were monitored for a minimum of 3 months [1,2,10]. Despite regular guideline-based follow-up, post-surgery or progression treatment decisions were made by a multidisciplinary team of urologists, radiation oncologists, and oncologists to tailor recommendations to individual patient needs [1,2].

### 2.2. Evaluating the Prognostic Impact and Predictors of Adverse Tumor Grade in Very Favorable Low- and Intermediate-Risk PCa Patients

The very favorable low- and intermediate-risk classes were characterized by PSA < 10 ng/mL, BPC < 50%, cT1c for both, and ISUP grade 1 for the former and 2 for the latter. Exclusion criteria included PSA 10–20 ng/mL, BPC > 50%, cT2a/b, and ISUP grade group 3. The primary aim was to assess the prognostic significance of adverse tumor grade concerning disease progression, defined as biochemical recurrence, local recurrence, and/or distant metastases. The secondary goal was to identify predictors of unfavorable tumor grade, defined as ISUP > 2. Clinical and pathological parameters were evaluated for study endpoints, excluding perioperative factors.

### 2.3. Statistical Analyses

Continuous variables were expressed as medians and interquartile ranges (IQR) and categorical ones as frequencies and percentages. The time between surgery and the occurrence of the clinical outcome of interest (PCa progression) or the last follow-up was recorded as the time to event occurrence. Univariate and multivariate Cox proportional hazards models were employed to evaluate the association of clinical and pathological factors with the risk of PCa progression, yielding hazard ratios and corresponding 95% confidence intervals (CI). Survival risk curves were generated accordingly. Additionally, the associations of clinical and pathological factors with unfavorable tumor grade (ISUP > 2) were examined using logistic regression models (both univariate and multivariate analyses). All statistical tests were performed using SPSS IBM SPSS Statistics version 26.0 (Armonk, NY, USA: IBM Corp.). All tests were two-sided with a significance level set at *p* < 0.05.

## 3. Results

### 3.1. Demography of Population and Subgroups including EAU Very Favorable Low- and Intermediate-Risk Classes

The study collected a population of 289 patients, including 178 low-risk cases (61.1%) and 111 intermediate-risk subjects (38.4%). Population demography is reported in the first part of Table 1. In the surgical samples, unfavorable tumor grade (ISUP > 2) was detected in 82 cases (28.4%), while adverse tumor stage, including ECE in 15 subjects SVI in 11 cases, was observed in 9% of patients. Overall, 59 patients (20.4%) exhibited surgical margins involved by cancer, with non-focal involvement noted in 12 (4.2%) cases. ePLND was performed in 101 cases (34.9%), with pelvic lymph node invasion identified in 3 (3.0%) patients. The median (IQR) follow-up was 49 months (24–67 months). In 25 cases (8.7%), patients underwent adjuvant androgen deprivation therapy, and RT was administered in 32 (11.1%), with 19 (6.6%) undergoing salvage treatment. Three deaths occurred (1%), of which one was PCa-related (0.3%).

Subgroup demography is illustrated in Table 2. The ISUP grade group at biopsy was the only clinical factor differentiating the two subsets. In the surgical samples, favorable intermediate-risk patients compared with low-risk cases showed a higher likelihood of larger tumors associated with adverse stage and grade; however, the latter was the only independent pathological factor differentiating the two groups. Intermediate-risk patients compared with low-risk cases underwent ePLND more frequently (59.9% vs. 19.7%) and had PLNI (4.5% vs. 0%); moreover, follow-up was longer for the latter, 54 months (36.7–71 months), than for the former, 38 months (19–70 months). Low-risk patients underwent ADT (7.9% vs. 9.9%) and salvage RT (5.1% vs. 9.9%) less frequently.

### 3.2. Prognostic Impact of Biopsy ISUP Grade Group 2 and Unfavorable Tumor Grade on PCa Progression

Overall, PCa progression was observed in 29 patients (10%). As depicted in Table 1, patients exhibited a greater likelihood of disease progression with biopsy ISUP grade group 2, adverse tumor grade (ISUP > 2), and positive surgical margins. Interestingly, pathology ISUP grade group 2 also displayed no association with progression. Excluding ISUP grade group 2, clinical factors demonstrated no association with PCa progression. As shown in Table 3 (multivariate analysis), adverse tumor grade and biopsy ISUP grade group 2 remained independent predictors of the risk PCa progression after adjusting for all parameters, with the former showing stronger associations (hazard ratio, HR = 4.478; 95% CI: 1.840–10.895; *p* = 0.001) than the latter (HR = 2.336; 95% CI: 1.057–5.164; *p* = 0.036).

**Figure 1:**
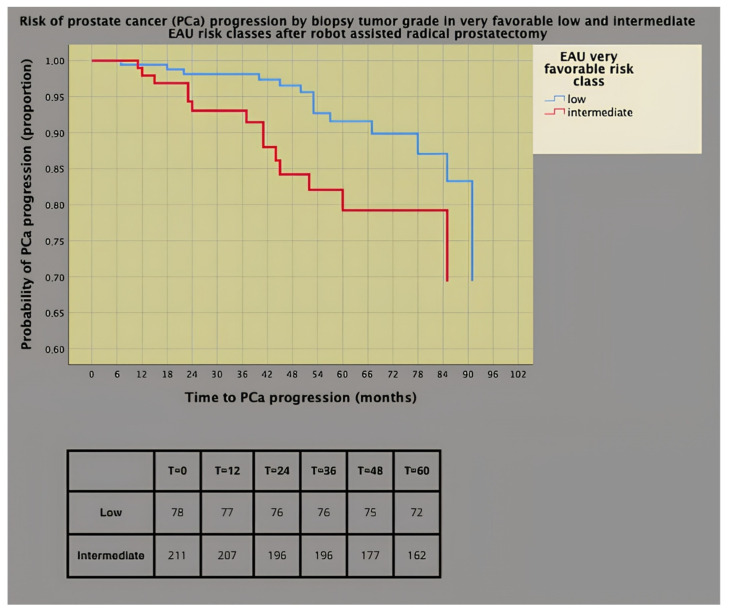
Survival risk curves of prostate cancer (PCa) progression (univariate Cox’s proportional hazards) stratified by International Society of Urologic Pathology (ISUP) grade group at diagnostic biopsy (hazard ratio, HR = 2.231; 95% CI: 1.072–4.643; *p* = 0.032).

**Figure 2:**
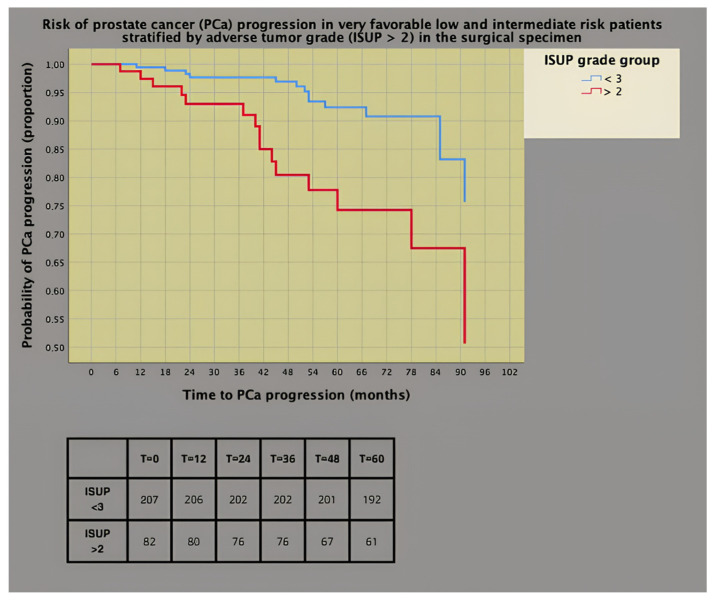
Survival risk curves of prostate cancer (PCa) progression (univariate Cox’s proportional hazards) stratified by adverse pathology tumor grade according to the International Society of Urologic Pathology (ISUP > 2; hazard ratio, HR = 3.300; 95% CI: 1.590–4.643; *p* = 0.001).

Figure 1 and Figure 2 show the survival risk curves of PCa progression (univariate Cox proportional hazards) for biopsy ISUP grade group 2 and adverse tumor grade (ISUP > 2) in the surgical specimen. As illustrated in Figure 1, the prognostic impact was worse for the ISUP grade group 2 than for group 1, with 22% of patients in the former group progressing at 5 years versus only 8% of the latter.

As shown in Figure 2, the prognostic impact was worse for adverse tumor grade than for favorable tumor grade (ISUP < 3), with 26% of patients in the former group progressing at 5 years versus only 8% of the latter.

Subgroup analysis confirmed that adverse tumor grade (ISUP > 2) was an unfavorable prognostic factor for PCa progression, as shown in Table 4 (univariate and multivariate models); however, the prognostic impact was stronger for the favorable intermediate-risk group (HR = 9.648; 95% CI: 1.962–47.449; *p* = 0.005) than for the low-risk category (HR = 6.283; 95% CI: 1.456–27.115; *p* = 0.014) after adjusting for all parameters.

**Figure 3:**
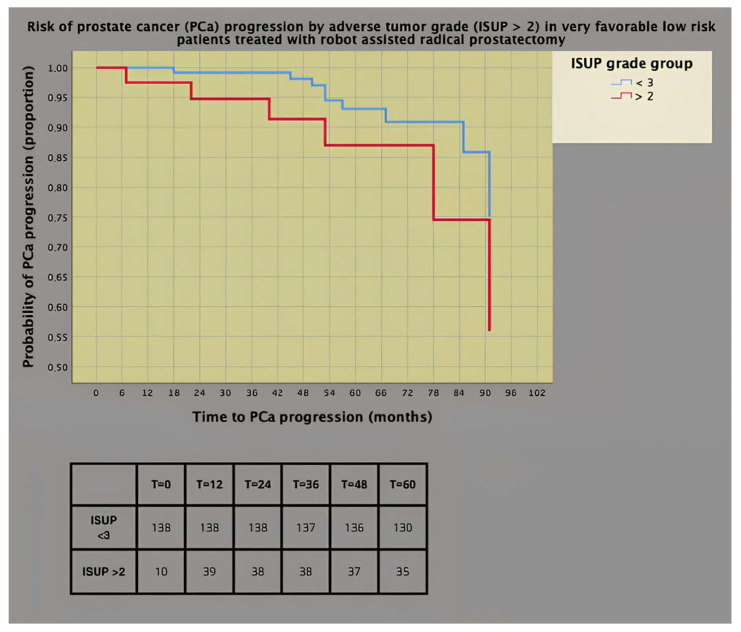
Survival risk curves of prostate cancer (PCa) progression (univariate Cox’s proportional hazards) stratified by adverse pathology tumor grade according to the International Society of Urologic Pathology (ISUP) system in very favorable low-risk PCa patients.

**Figure 4:**
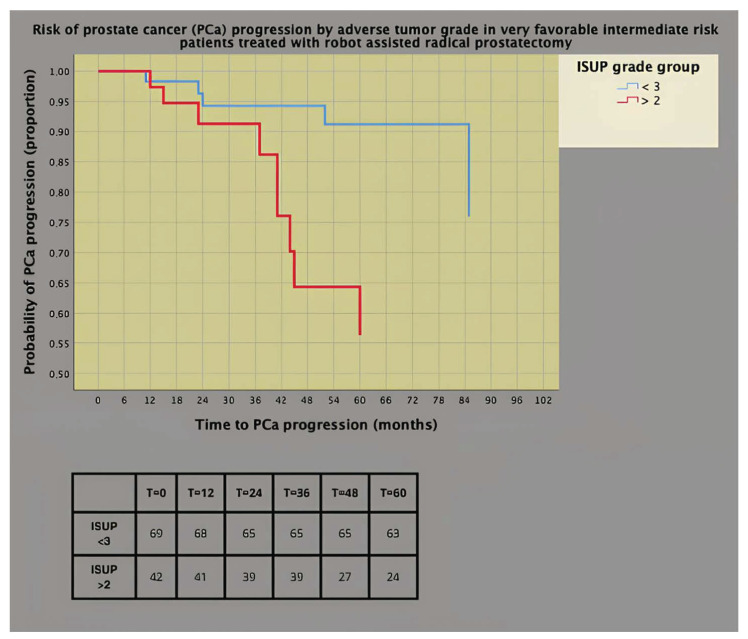
Survival risk curves of prostate cancer (PCa) progression (univariate Cox’s proportional hazards) stratified by adverse tumor grade according to the International Society of Urologic Pathology in very favorable intermediate-risk PCa patients.

Survival risk curves for PCa progression (univariate Cox proportional hazards) stratified by pathology tumor grade in very favorable low- and intermediate-risk groups are illustrated in Figure 3 and Figure 4, respectively. In the former subgroup, as shown in Figure 3, the prognostic impact was worse for adverse tumor grade (ISUP > 2) than for favorable grade (ISUP < 3), with 13% of patients in the former progressing at 5 years versus only 7% in the latter (HR = 5.590; 95% CI: 1.124–27.797; *p* = 0.035).

In the latter subgroup, as depicted in Figure 4, the prognostic impact was worse for adverse tumor grade (ISUP > 2) than for favorable pathology grade (ISUP < 3), with 44% of patients in the former progressing at 5 years versus only 6% in the latter (HR = 3.869; 95% CI: 1.286–11.637; *p* = 0.016).

### 3.3. Associations of Unfavorable Tumor Grade with Clinical and Pathological Factors

The relationships of adverse tumor grade with clinical and other pathological factors are reported in Table 5, indicating that patients with an adverse tumor grade, associated with larger tumors having an adverse stage and positive surgical margins, were more likely to be older, to have higher PSAD levels, and to belong to the intermediate-risk group (biopsy ISUP grade group 2); however, on multivariate analysis, only older age and biopsy ISUP grade group 2 were independent clinical predictors of pathology adverse tumor grade, associated with larger tumors presenting adverse stage.

Table 6 shows that, in the very favorable EAU low-risk class, older age and PSAD ≥ 0.15 ng/mL were independent clinical predictors of adverse tumor grade (ISUP > 2), which was associated with larger tumors, presenting more likely with adverse stage. In the very favorable intermediate-risk class, there were no significant clinical and pathological predictors of adverse tumor grade.

## 4. Discussion

In the natural course of prostate cancer (PCa), biochemical recurrence following primary surgery or radiation signifies adverse oncological outcomes, preceding metastatic progression, ultimately impacting disease-specific and overall mortality. Consequently, unfavorable tumor grade in the surgical specimen serves as the primary indicator of PCa progression [1,2,3]. So far, tumor grade misclassification in early PCa, including very favorable subsets of the low- and intermediate-risk classes, serves as the primary predictor of disease progression and, consequently, is a serious issue for clinicians when making treatment decisions [1,2,13,14]. The new grading group system proves to be a strong predictor of PCa progression in a large cohort of 20,845 cases. Higher-grade groups show significantly increased hazard ratios compared to grade group 1 (HR 2.2, 7.3, 12.3, and 23.9 from ISUP to 5, respectively). Grade group 2 indicates a favorable prognosis with rare metastases. However, the study does not stratify the primary endpoint based on clinical prognostic risk groups [15].

However, adverse tumor pathology affects prognosis differently across risk groups, with better outcomes in low-risk compared to intermediate- and high-risk categories. Treatment options may involve delayed intervention for low- and favorable intermediate-risk PCa. According to the Cambridge Prognostic Group (CPG) system, the 10-year risk of death for PCa is 1.2% for low-risk disease (CPG 1) and 2.3% for favorable intermediate-risk PCa (CPG 2), including ISUP grade group 2 or PSA levels of 10–20 ng/mL and stages T1-T2. Untreated, these rates increase to 4.2% and 4.7%, respectively [16,17]. A recent North American study, evaluating rates of adverse surgical pathology with survival implications in favorable intermediate-risk patients compared with low-risk cases, showed that adverse pathology after RP was observed was three times more prevalent in the former group compared to the latter. Additionally, the latter group demonstrated superior overall survival [18]. In the low- and intermediate-risk categories, a recent comprehensive literature review highlighted that unfavorable pathology affects up to 30% of cases and contributes to disease progression. Furthermore, disease progression might also be anticipated by a short PSA doubling time (PSA-DT) and high tumor grade post-surgery [19].

In a large European cohort, a study found that a PSA-DT < 2 years and ISUP > 3 identified a high-risk group linked to metastatic progression and cancer-specific mortality. However, the study included ISUP grade group 3 in the low-risk category. Additionally, researchers suggested refining EAU BCR groups [20]. Another multicenter study revealed that 10.6% of intermediate-risk patients had favorable pathology (ISUP grade group 1), associated with better PSA recurrence-free survival rates compared to unfavorable pathology (94.2% vs. 74.4% at 4 years) [21]. However, this study had limitations, including a short follow-up and lack of central pathology revision.

Moreover, while salvage RARP is feasible, albeit technically challenging, after recurrence after primary treatment, including ADT, no data regarding patients with low or favorable intermediate risk who then underwent these subsequent treatments are available to assess the impact of ADT on PCa progression [22,23,24,25,26].

Our results showed that adverse tumor grade was an independent negative prognostic factor in a highly selected population, including EAU low- and intermediate-risk classes, which closely resembled each other, in including PSA < 10 ng/mL, clinical stage cT1c, BPC < 50%, but differed only in biopsy ISUP, defined as 1 for the former and 2 for the latter. Therefore, 5-year progression rates were higher in subjects hiding adverse pathology (ISUP > 2) than in cases without, 26% vs. 8%, respectively; however, the prognostic impact was not the same among the very favorable, low- and intermediate-risk classes; accordingly, 5-year progression rates were 44% for the latter and only 13% for the former. Interestingly, pathology ISUP grade group 2 compared with group 1 did not associate with PCa progression, thus showing that its detection has no negative prognostic impact in these highly selected sets of patients. This information is important for clinicians (urologists and radiation oncologists) when counseling patients; moreover, it supports the current guidelines on recommending AS for ISUP grade group 2 (see reference literature by EAU and NCCN) [1,2].

Additionally, a European multicenter study investigated the impact of clinical criteria on predicting adverse disease outcomes in individuals with low and intermediate PCa risk treated with RP. Results showed that PSA levels, PSAD, tumor grade group, and tumor stage were all independent predictors of unfavorable pathology. Specifically, unfavorable pathology was observed in 17.9% of cases within the low-risk category and 44.3% in the intermediate-risk category. However, the study’s utility is limited by its inclusion criteria and lack of centralized pathology review due to its multicenter nature [4]. In a retrospective analysis, a favorable intermediate-risk subgroup was characterized by ISUP grade 2 with PSA levels below 20 ng/mL and no more than two positive biopsy cores. Adverse pathology was detected in approximately 25% of specimens, with unfavorable tumor grade (ISUP > 2) accounting for 23.2% of cases. In addition, they showed that PSA and PSAD levels were associated with unfavorable pathology; however, biopsy ISUP 1 was not available for analysis, and they were unable to clinically identify a subgroup with adverse pathology findings with rates comparable to those of low-risk patients [27]. Recently, a study in North America crafted and validated a nomogram forecasting adverse pathology, delineated as ISUP > 1 and/or ECE/SVI, in low-risk patients who underwent surgical intervention; the model, integrating conventional clinical factors with mpMRI findings, demonstrated an accuracy rate of 87%. However, the study faced several limitations owing to its retrospective nature, the involvement of only one high-volume surgeon, and the omission of considering the distinction between favorable and unfavorable tumor upgrading, which could potentially yield differing prognostic implications [28].

In a very favorable subgroup of low- and intermediate-risk PCa patients treated with RP, we demonstrated that adverse tumor grade in the surgical specimen resulted in an unfavorable prognostic impact on the population and both subgroups, with the latter significantly worse than the former; as a result, adverse tumor grade rates were higher for the latter than for the former (37.8% vs. 22.5%), and the same pattern was also observed for adverse tumor stage (13.5% vs. 6.2%). Our results demonstrated that ISUP grade group 2 at biopsy was an independent clinical predictor of tumor misclassification, thus clustering the two subgroups that were closely related to each other for the remaining clinical factors; furthermore, our investigation showed that older patients and those with PSAD ≥ 0.15 ng/mL/mL were more likely to have tumor misclassification in the very low-risk subset, thus representing further parameters for stratifying subjects. Unfortunately, we were unable to identify clinical predictors of adverse tumor grade other than ISUP grade 2 in the very favorable intermediate-risk group, thus suggesting that biomolecular markers are needed to select unfavorable clusters.

Tumor misclassification poses a challenge in monitoring patients with low- and favorable intermediate-risk profiles for deferred treatment. In fact, AS exhibits rising adoption rates among intermediate-risk patients in North America, especially among the elderly, with favorable intermediate PCa risk. Furthermore, it has been shown to be a safer option for the favorable class compared to the unfavorable group, with the latter demonstrating significantly higher prostate cancer-specific mortality rates than other active treatments, including RP and radiation therapy [29]. While individuals classified within the PCa low- and favorable intermediate-risk categories may contemplate deferred treatment strategies, it is important to note that their prognoses are not equivalent [1,2,3]. Therefore, although the utilization of AS for patients with favorable intermediate-risk disease is on the rise in North America, additional follow-up and research are warranted to assess safety and efficacy [29].

While the aforementioned EAU criteria for the favorable intermediate-risk class are very selective, NCCN criteria are less selective, due to the inclusion of all of the following intermediate-risk factors, as grade group 1 or 2, less than 50% of positive biopsy cores [1,2,3]. Currently, additional clinical classification parameters are needed to improve the monitoring and management of very favorable low- and intermediate-risk patients [6,7,30,31].

In our analysis, we selected very favorable subsets of low- and intermediate-risk patients presenting in daily practice; as a result, unfavorable tumor grade is to be expected in less than one-third of patients; however, those presenting with favorable clinical parameters but ISUP 2 on biopsy were more likely to have higher unfavorable tumor upgrading (37.8% vs. 22.5%) and upstaging (13.5% vs. 6.2%) rates. This additional information may assist urologists and radiation oncologists in making informed treatment decisions when advising these specific patient subsets. Thus far, very favorable low- and intermediate-risk patients defined according to the parameters of this study, despite having criteria that elect them to AS, still have the risk of delayed treatment for both subgroups, but which is certainly more unfavorable for the latter. On the other hand, very favorable intermediate-risk patients who underwent RP and with an adverse tumor grade in the surgical specimen need close follow-up monitoring; furthermore, biochemical persistence or early biochemical recurrence may indicate disease persistence or recurrence, which needs to be investigated with more advanced imaging modalities, such as PET-PSMA, to plan appropriate treatments. Moreover, when the same patient category is treated with primary radiation, adjuvant androgen deprivation therapy should be considered to avoid biochemical recurrence, which precedes disease progression and requires more extensive investigation, such as PET-PSMA, to provide further and more appropriate treatments. Overall, beyond treatments, patients should be informed that, although they belong to very favorable categories, there is a risk of tumor-grade misclassification, which is not negligible but certainly worse for the intermediate-risk group. In the favorable low-risk category under AS, older patients presenting with PSAD ≥ 0.15 ng/mL/mL should be informed that they are at increased risk of adverse tumor grade misclassification and, consequently, at risk of delayed treatment. These are some practical examples of how this information may help physicians in clinical decision-making, but more applications may occur, especially when trying to personalize treatment even in the older patient population.

Explanations are needed to interpret our findings, which indicate that tumor misclassification served as a negative prognostic factor for disease progression and was associated with clinical factors including ISUP grade group 2, older age, and PSAD ≥ 0.15 ng/mL/mL. Theoretically, older patients, in comparison to younger individuals, are more prone to longer exposure to genetic mutation dynamics and may also have a compromised immune system, which could contribute to the development of unfavorable tumor grades. Additionally, higher PSAD levels could be associated with more aggressive and extensive tumors growing in smaller prostates. Lastly, high-grade cancer cell populations may exhibit a high-density growth pattern, potentially intensified by local stimulating factors. However, these hypotheses require verification through controlled studies.

We acknowledge several limitations in our study. Firstly, it was retrospective and conducted at a single center and included only patients who were ultimately submitted to RARP, potentially limiting the generalizability of the findings. A power analysis to determine sample-size adequacy was not conducted in this retrospective cohort study. Consequently, the study’s ability to detect significant differences may be limited. Additionally, we did not assess mpMRI and molecular/genetic tests due to their unavailability in all cases. Moreover, conducting further analyses that encompass various comorbidities could evaluate their potential impacts on cancer progression during RARP. Furthermore, overall and cancer-specific survival were not evaluated due to the limited number of events. We also did not evaluate the percentage of cancer involvement in each biopsy core and the percentage of Gleason pattern 4 in biopsy-positive cores due to data unavailability, and we were not able to centrally review all specimens at prostate biopsy. A further possible drawback in the study is that it was not possible to employ multiple pathologists to reassess the specimens in order to reduce possible grading errors.

However, our study has strengths. The primary outcome was assessed at disease progression, a robust endpoint. The follow-up length was adequate, and procedures were conducted by both low- and high-volume surgeons, minimizing bias. Lastly, all surgical specimens were evaluated by our dedicated high-volume pathologist, ensuring consistency and accuracy in pathological assessments.

## 5. Conclusions

In a very favorable PCa patient population, including low- and intermediate-risk cases that differed only in the ISUP grade group, adverse tumor grade was an unfavorable prognostic factor for disease progression, but the impact was more negative for the latter than the former due to higher rates of PCa progression; as a result, almost one in three patients harbored adverse tumor grades with higher rates for the latter than the former. Active surveillance in very favorable intermediate-risk patients is still a hazard, so molecular and genetic testing should be performed on biopsy specimens to cluster aggressive tumors leading to an unfavorable tumor grade in the surgical specimen.

## Figures and Tables

**Table 1:** Risk of prostate cancer (PCa) progression in 289 very favorable EAU low- and intermediate-risk patients (*) treated with robot-assisted radical prostatectomy (RARP).

Number (%)	Population	No. PCa Progression	PCa Progression	Univariate Analysis	*p*-Value
	289	260 (90%)	29 (10%)	HR (95% CI)	
**Clinical factors**					
Age (years)	65 (59–69)	65 (59–69)	64 (58.5–68)	1.003 (0.946–1.063)	0.923
BMI (kg/m^2^)	25.9 (24–27.7)	26.1 (24–27.8)	24.8 (24–26)	0.884 (0.769–1.016)	0.082
PV (mL)	40 (30–51)	40 (30–50)	39 (28.5–52)	1.003 (0.983–1.023)	0.790
PSA (ng/mL)	5.9 (4.6–7.5)	5.9 (4.6–7.4)	6.2 (4.6–7.6)	1.047 (0.866–1.267)	0.634
PSAD < 0.15 (ng/mL/cc)	148 (51.2)	134 (51.5)	14 (48.3)	Ref	
PSAD ≥ 0.15 (ng/mL/cc))	141 (48.8)	126 (48.5)	15 (51.7)	1.095 (0.528–2.273)	0.807
BPC (%)	21 (14–31)	21 (14–30)	25 (14.5–33)	1.010 (0.979–1.041)	0.540
ISUP = 1	178 (61.6)	163 (62.7)	15 (51.7)	Ref	
ISUP = 2	111 (38.4)	97 (37.3)	14 (48.3)	**2.231 (1.072–4.643)**	**0.032**
**Pathological factors**					
ISUP = 1	78 (27)	75 (28.8)	3 (10.3)	Ref	
ISUP = 2	129 (44.6)	118 (45.8)	11 (37.9)	2.812 (0.784–10.087)	0.113
ISUP > 2	82 (28.4)	67 (25.8)	15 (51.7)	**6.691 (1.933–23.158)**	**0.003**
PW (gr)	52 (42.5–65)	52 (43–63.8)	55 (40–74)	1.007 (0.990–1.025)	0.433
TL (%)	12.5 (7.7–20)	10 (5.6–20)	15 (10–20)	0.996 (0.963–1.030)	0.803
pT2	263 (91)	240 (92.3)	23 (79.3)	Ref	
EPE (ECE or SVI)	26 (9)	20 (7.7)	6 (20.7)	2.264 (0.919–5.580)	0.076
R0	230 (79.6)	211 (81.2)	19 (65.5)	Ref	
R1	59 (20.4)	49 (18.8)	10 (34.5)	**2.394 (1.110–5.164)**	**0.026**

Legend: continuous variables are reported as medians (IQR, interquartile ranges) and categorical factors as frequencies (percentages); HR, hazard ratio; CI: confidence interval; see Materials and Methods for abbreviations; (*) defined as: PSA < 10 ng/mL, BPC < 50%, cT1c, ISUP = 1 (very favorable low risk) or = 2 (very favorable intermediate risk).

**Table 2:** Demographic characteristics of a cohort comprising 289 prostate cancer (PCa) patients, including those classified under the very favorable EAU low- and intermediate-risk classes (*), who underwent robot-assisted radical prostatectomy (RARP).

Number (%)	Very Favorable Low-Risk PCa	Very Favorable Intermediate-Risk PCa	Univariate Analysis	*p*-Value	Multivariate Analysis	*p*-Value
	178 (61.6)	111 (38.4)	OR (95% CI)		OR (95% CI)	
Age (years)	65 (59–70)	64 (60–69)	0.996 (0.960–1.033)	0.829	0.986 (0.947–1.026)	0.478
BMI (kg/m^2^)	26.1 (24–27.8)	25.2 (23.9–27.3)	0.953 (0.876–1.037)	0.266	0.939 (0.860–1.026)	0.163
PV (mL)	40 (30.7–50.5)	40 (30–50)	0.993 (0.980–1.007)	0.341	1.001 (0.977–1.026)	0.922
PSA (ng/mL)	5.9 (4.4–7.6)	5.9 (4.6–7.4)	1.023 (0.904–1.158)	0.717	0.925 (0.777–1.101)	0.380
PSAD < 0.15 (ng/mL/cc)	98 (55.1)	134 (51.5)	Ref		Ref	
PSAD ≥ 0.15 (ng/mL/cc))	80 (44.9)	126 (48.5)	1.494 (0.928–2.407)	0.098	1.704 (0.799–3.637)	0.168
BPC (%)	20 (14–31)	21 (14–30)	1.006 (0.985–1.028)	0.586	1.004 (0.981–1.027)	0.758
ISUP < 3	138 (77.5)	69 (62.2)	Ref		Ref	
ISUP > 2	40 (22.5)	42 (37.8)	**2.100 (1.248–3.534)**	**0.005**	**1.849 (1.053–3.249)**	**0.032**
PW (gr)	52 (42–65)	54 (45–63.5)	0.999 (1.987–1.011)	0.889	1.009 (0.989–1.029)	0.378
TL (%)	10 (5–20)	15 (10–20)	**1.026 (1.004–1.045)**	**0.019**	1.023 (1.000–1.047)	0.052
pT2	167 (93.8)	96 (86.5)	Ref		Ref	
pT3	11 (6.2)	15 (13.5)	**2.372 (1.047–5.373)**	**0.038**	1.820 (0.749–4.422)	0.186
R0	142 (79.8)	88 (79.3)	Ref		Ref	
R1	36 (20.2)	23 (20.7)	1.031 (0.537–1.854)	0.919	0.685 (0.349–1.344)	0.272

Legend: continuous variables are reported as medians (IQR, interquartile ranges) and categorical factors as frequencies (percentages); OR, odds ratio; CI: confidence interval; see Materials and Methods for abbreviations; (*) defined as: PSA < 10 ng/mL, BPC < 50%, cT1c, ISUP = 1 (very favorable low risk) or = 2 (very favorable intermediate risk).

**Table 3:** Multivariate analysis of the negative prognostic impact of unfavorable tumor grade (ISUP > 2) and biopsy grade group 2 on prostate cancer progression in 289 very favorable EAU low- and intermediate-risk patients (*) who underwent robot-assisted radical prostatectomy (RARP).

Endpoints	Adverse Tumor Grade (ISUP > 2)		Biopsy ISUP Tumor Grade 2 (**)	*p*-Value
Statistics	HR (95% CI)	*p*-Value	HR (95% CI)	
Hazard ratios adjusted for clinical factors	3.953 (1.733–9.017)	0.001	2.379 (1.109–5.103)	0.026
Hazard ratios adjusted for pathological factors	3.907 (1.704–8.961)	0.001	2.321 (1.094–4.925)	0.028
Hazard ratios adjusted for clinical and pathological factors	4.478 (1.840–10.895)	0.001	2.366 (1.057–5.164)	0.036

Legend: ISUP, International Society of Urologic Pathology prostate cancer tumor grading system; EAU, European Association of Urology; HR, odds ratio; CI: confidence interval; (*): defined as PSA < 10 ng/mL, BPC < 50%, cT1c, ISUP = 1 (very favorable low-risk) or = 2 (very favorable intermediate-risk); (**), reference biopsy ISUP grade group 1; see also Table 1.

**Table 4:** Prostate cancer (PCa) progression: impact of adverse tumor grade (ISUP > 2) in the surgical specimen of very favorable EAU low- and intermediate-risk patients treated with robot-assisted radical prostatectomy (univariate and multivariate analysis).

Statistics	Very Favorable Low-Risk PCa (*)	*p*-Value	Very Favorable Intermediate-Risk Pca (*)	*p*-Value
	HR (95% CI)		HR (95% CI)	
Unadjusted hazard ratios	5.590 (1.124–27.797)	0.035	3.869 (1.286–11.637)	0.016
Hazard ratios adjusted for clinical factors	6.352 (1.094–36.888)	0.039	5.300 (1.505–18.859)	0.009
Hazard ratios adjusted for pathological factors	5.947 (1.737–20.064)	0.004	4.451 (1.275–15.530)	0.019
Hazard ratios adjusted for clinical and pathological factors	6.283 (1.456–27.115)	0.014	9.648 (1.962–47.499)	0.005

Legend: ISUP, International Society of Urologic Pathology prostate cancer tumor grading system; EAU, European Association of Urology; HR, odds ratio; CI: confidence interval; (*): defined as PSA < 10 ng/mL, BPC < 50%, cT1c, ISUP = 1 (very favorable low-risk) or ISUP = 2 (very favorable intermediate-risk); see also Table 1.

**Table 5:** Associations of clinical and pathological factors with unfavorable tumor grade (ISUP > 2) in 289 very favorable low- and intermediate-risk prostate cancer patients treated with robot-assisted radical prostatectomy.

Factors	Favorable Tumor Grade (ISUP < 3)	Unfavorable Tumor Grade (ISUP > 2)	Univariate Analysis	*p*-Value	Multivariate Analysis	*p*-Value
Number (%)	207 (71.6)	82 (28.4)	OR (95% CI)		OR (95% CI)	
Age (years)	64 (58–68)	67 (62–70.2)	**1.065 (1.021–1.112)**	**0.004**	**1.077 (1.028–1.129)**	**0.002**
BMI (kg/m^2^)	26 (23.9–27.7)	25.4 (24.1–28.4)	1.019 (0.998–1.114)	0.672	1.041 (0.942–1.150)	0.432
PSA (ng/mL)	5.8 (4.5–7.2)	6.2 (4.6–7.9)	1.104 (0.965–1.263)	0.151	1.067 (0.875–1.301)	0.522
PV (mL)	40 (30–52)	40 (29.7–47.2)	0.996 (0.970–1.001)	0.075	0.988 (0.959–1.018)	0.417
BPC (%)	21 (14–31)	21.2 (14–33)	1.013 (0.990–1.037)	0.266	1.008 (0.983–1.035)	0.528
PSAD < 0.15 (ng/mL/cc)	115 (55.6)	33 (40.2)	Ref		Ref	
PSAD ≥ 0.15 (ng/mL/cc)	92 (44.4)	49 (50.8)	**1.856 (1.104–3.121)**	**0.020**	1.174 (0.492–2.801)	0.717
ISUP = 1	138 (66.7)	40 (48.8)	Ref		Ref	
ISUP = 2	69 (33.3)	42 (51.2)	**2.100 (1.284–3.534)**	**0.005**	**1.903 (1.080–3.354)**	**0.026**
PW (gr)	53 (44–67)	50 (40–60.2)	0.994 (0.981–1.018)	0.409	1.003 (0.980–1.027)	0.791
TL (%)	10 (5–20)	16.5 (10–25)	**1.035 (1.014–1.057)**	**0.001**	**1.028 (1.004–1.053)**	**0.024**
pT2	197 (95.2)	66 (80.5)	Ref		Ref	
pT3	10 (4.8)	16 (19.3)	**4.776 (2.066–11.038)**	**<0.0001**	**2.646 (1.071–6.538)**	**0.035**
R0	171 (82.6)	59 (72)	Ref		Ref	
R1	36 (17.4)	23 (28)	**1.852 (1.015–3.378)**	**0.045**	1.192 (0.586–2.424)	0.627

Legend: continuous variables are reported as medians (IQR, interquartile ranges) and categorical factors as frequencies (percentages); OR, odds ratio; CI, confidence interval; see also Materials and Methods for abbreviations.

**Table 6:** Associations of clinical and pathological factors with unfavorable tumor grade (ISUP > 2) in 178 very favorable low-risk prostate cancer patients (*) treated with robot-assisted radical prostatectomy.

Factors	Favorable Tumor Grade (ISUP < 3)	Unfavorable Tumor Grade (ISUP > 2)	Univariate Analysis	*p*-Value	Multivariate Analysis (**)	*p*-Value
Number (%)	138 (77.5)	40 (22.5)	OR (95% CI)		OR (95% CI)	
Age (years)	64 (58–68)	67.5 (62.2–71)	**1.084 (1.020–1.153)**	**0.009**	**1.078 (1.008–1.154)**	**0.029**
BMI (kg/m^2^)	26.2 (24.2–27.8)	25.5 (23.7–28.2)	0.983 (0.867–1.115)	0.975		
PSA (ng/mL)	5.6 (4.3–7.1)	7 (4.4–8.8)	**1.226 (1.026–1.465)**	**0.025**		
PV (mL)	42 (31.8–52.2)	40 (30–46.5)	**0.972 (0.947–0.997)**	**0.029**		
PSAD < 0.15 (ng/mL/cc)	86 (62.3)	12 (30)	Ref			
PSAD ≥ 0.15 (ng/mL/cc)	52 (37.7)	28 (70)	**3.859 (1.807–8.242)**	**<0.0001**	**3.077 (1.377–6.879)**	**0.006**
BPC (%)	20 (14–29.5)	21.2 (14–33)	1.016 (0.984–1.049)	0.334		
PW (gr)	52.7 (43.5–69.1)	49.5 (42–52.7)	0.987 (0.968–1.007)	0.198		
TL (%)	10 (5–15)	16.5 (10–25)	**1.040 (1.009–1.072)**	**0.012**	**1.041 (1.007–1.075)**	**0.016**
pT2	135 (97.8)	32 (80)	Ref			
pT3	3 (2.2)	8 (20)	11.250 (2.825–44.795)	**0.001**	**4.509 (4.003–20.275)**	**0.045**
R0	115 (83.3)	27 (67.5)	Ref			
R1	23 (16.7)	13 (32.5)	**2.407 (1.083–5.352)**	**0.031**		

Legend: continuous variables are reported as medians (IQR, interquartile ranges) and categorical factors as frequencies (percentages); OR, odds ratio; CI, confidence interval; (*), defined as PSA < 10 ng/mL, BPC < 50%, cT1C, ISUP = 1 at biopsy; (**), by Wald’s forward method for the high correlation between PV, PSA, PSAD; see also Materials and Methods for abbreviations.

## Data Availability

The data presented in this study are available on reasonable request from the corresponding author.
